# Single-Cell Transcriptomic Analysis Unveils Key Regulators and Signaling Pathways in Lung Adenocarcinoma Progression

**DOI:** 10.3390/biomedicines13071606

**Published:** 2025-06-30

**Authors:** Jialu Ma, Caleb McQuay, John Talburt, Amit K. Tiwari, Mary Qu Yang

**Affiliations:** 1 MidSouth Bioinformatics Center and Joint Bioinformatics Graduate Program, University of Arkansas at Little Rock, Little Rock, AR 72204, USA; 2 Information Science, University of Arkansas at Little Rock, Little Rock, AR 72204, USA; 3 Computer Science, University of Arkansas at Little Rock, Little Rock, AR 72204, USA; 4 Department of Pharmaceutical Sciences, College of Pharmacy, University of Arkansas for Medical Sciences, Little Rock, AR 72205, USA; atiwari@uams.edu

**Keywords:** lung adenocarcinoma progression, single-cell RNA sequencing, transcription regulator, signaling pathways

## Abstract

**Background:** Lung adenocarcinoma (LUAD) remains a leading cause of cancer-related mortality despite advances in treatments, necessitating more effective therapeutic strategies. Single-cell RNA sequencing (scRNA-seq) technology has revolutionized our ability to dissect the cellular complexity of cancers, which is often obscured in conventional bulk transcriptomic experiments. **Methods:** In this study, we performed an integrative analysis of scRNA-seq data from multiple LUAD patient cohorts to investigate cell-type-specific transcriptomic changes across disease stages. Clustering, lineage trajectory analysis, and transcriptional regulatory network reconstruction were employed to identify stage-specific gene markers and their upstream regulators. Additionally, we constructed intercellular communication networks to evaluate signaling changes within the tumor microenvironment (TME) during LUAD progression. **Results:** Our analysis revealed that epithelial cells from stage IV tumors exhibited a distinct transcriptional profile compared to earlier stages, a separation not observed in immune or stromal cell populations. We identified a panel of gene markers that differentiated epithelial cells across disease stages and effectively stratified patients into subgroups with distinct survival outcomes and TME compositions. Regulatory network analysis uncovered key transcription factors, including ATF3, ATF4, HSF1, KLF4, and NFIC, as potential upstream regulators of these stage-specific genes. Moreover, cell–cell communication analysis revealed a significant increase in signaling originating from epithelial cells and a concomitant decrease in immune-derived signals in late-stage LUAD. We identified several signaling pathways enriched in stage-specific crosstalk, including Wnt, PTN, and PDGF pathways, which may play critical roles in LUAD progression. **Conclusions:** This study provides a comprehensive single-cell resolution map of LUAD progression, highlighting epithelial-driven regulatory programs and dynamic intercellular communication within the TME. Our findings uncover novel molecular markers and regulatory mechanisms with potential prognostic and therapeutic value for more precise treatment.

## 1. Introduction

Lung carcinoma is a leading cause of cancer-related mortality worldwide, with an estimated 18.4% of all cancer deaths in 2018 [1]. Lung cancer is classified into small-cell lung (SCLC) and non-small-cell lung cancers (NSCLC), the latter representing about 80–85% of total cases [2]. NSCLC can be further classified into adenocarcinoma and squamous cell carcinoma. Only around 20% of lung cancer patients are eligible for curative surgery, yet up to 80% of those who undergo resection experience postoperative recurrence [3]. Moreover, roughly one-third of NSCLC cases are diagnosed at stage III, where 5-year survival falls from 36% in stage IIIA to 13% in stage IIIC [4]. Among the subtypes of NSCLC, lung adenocarcinoma (LUAD) is the most prevalent. Major risk factors for LUAD include smoking, environmental exposure, and genetic predispositions. As smoking rates decline in the population [5], there has been a marked increase in the proportion of patients with lung cancer who have never smoked, particularly among younger people and women [6]. Nonsmoking lung cancer patients are frequently diagnosed with LUAD [7,8]. Despite improvements in early diagnosis and treatment, patients often experience relapses and metastases. Consequently, the prognosis and overall survival rate of LUAD remain very poor [9,10].

Conventional treatment for LUAD includes surgery, chemotherapy, and radiotherapy. Owing to discoveries in molecular translation and clinical research, newer treatment approaches such as immunotherapy and targeted therapy have emerged as important options, which offer improved survival rates and quality of life for many patients with LUAD. For example, immune checkpoint blockade has become a significant treatment method for various cancers, including LUAD. In particular, inhibitors of the programmed cell death protein-1/programmed cell death ligand-1 (PD-1/PD-L1) enhance the antitumor activity of cytotoxic T cells and have been proven to be effective in the treatment of locally advanced LUAD [11,12]. Tyrosine kinase inhibitors targeted at known oncogenic driver mutations, such as EGFR and BRAF gene mutations [13,14] and ALK and ROS1 fusions [15,16,17], demonstrate efficacy in treating patients harboring these mutations [18]. However, patient responses to targeted therapy and immunotherapy vary widely. While some patients achieve a complete response, others may experience partial responses, and many may not respond at all. Furthermore, tumor progression can still occur in patients who initially respond to treatment, often due to the development of acquired resistance [19]. The inherent heterogeneity of LUAD further complicates the efficacy and durability of these treatments. To address these challenges, preclinical models such as patient-derived xenografts (PDX) have been used to evaluate therapeutic responses in a more physiologically relevant context [20,21]. However, they often lack resolution at the single-cell level.

Single-cell sequencing technology, which allows the assessment of individual cells in the tumor microenvironment (TME), provides opportunities to gain novel insights into disease progression and cellular heterogeneity, potentially leading to new treatment strategies. LUAD originates mainly from epithelial cells located in the distal part of the lung [22]. Oncogenic mutations in pulmonary epithelial cells can cause uncontrolled cellular proliferation, contributing to the development of lung cancer [23,24]. Lung epithelial cells have self-renewal capacity and can regenerate other types of lung cells in response to injury and disease [25]. Dysregulation of inflammation has been reported to facilitate the transition of epithelial cells to mesenchymal cells, a process critical to the development of chronic obstructive pulmonary disease and lung cancer [26,27]. However, the molecular mechanisms underlying the progression of LUAD at the cellular level remain largely uncharacterized.

In this study, we analyzed the transcriptomic alterations of various cell types and their intercellular communications within the TME during the progression of LUAD by incorporating multiple single-cell RNA sequencing (scRNA-seq) datasets. We discovered a clear separation between stage IV LUAD epithelial cells and those from earlier stages, a distinction not seen in immune and stromal cell populations, as demonstrated through both cell clustering and lineage analysis. This unique separation pattern allowed us to extract a set of differentially expressed genes in epithelial cells that may serve as gene markers for LUAD progression at the cellular level. Given the inherent complexity and heterogeneity of LUAD, as observed in many other cancers, we expanded our analysis to multiple independent LUAD patient cohorts. We found that the expression of these gene markers robustly segregated LUAD patients into subgroups with distinct survival outcomes and TME compositions. To investigate the regulation of these gene signatures, we constructed transcriptional regulatory networks and identified their master regulators. Furthermore, we inferred cell-cell communication networks within the TME to assess changes in cellular interactions in response to LUAD progression. Our analysis revealed significantly increased communication signals originating from epithelial cells, particularly in the crosstalk between epithelial and fibroblast cells; in contrast, communication from immune cells notably decreased in late-stage LUAD. We also identified several key signaling pathways associated with the dynamics of the intercellular communication networks during LUAD progression. These findings present promising cellular-level biomarkers and drug targets, offering more precise opportunities for managing disease progression and developing improved treatment strategies for better clinical outcomes in patients.

## 2. Materials and Methods

### 2.1. LUAD Datasets

We obtained two scRNA-seq datasets, GSE131907 [28] and GSE127465 [29], from Gene Expression Omnibus (GEO) for this study. The GSE131907 dataset was generated using the 10× Genomics Single Cell 3′ platform, with libraries prepared by the Single Cell 3′ library and Gel Bead Kit V2, and sequenced on the Illumina HiSeq 2500 [28]. The sequencing reads were aligned to the GRCh38 human genome. For our study, we utilized cells derived from 26 lung tissue samples, including 11 normal and 15 tumor samples spanning stages I to IV. The GSE127465 dataset was generated using the inDrop platform. Libraries were prepared on the Illumina NextSeq platform and mapped to the GRCh38 human genome [29]. A total of 29,313 cells from tumor tissue samples collected from five LUAD patients across stages I, III, and IV were included in our study. The detailed metadata for GSE131907 and GSE127465 are provided in Supp. Table S1.

Additionally, we obtained four LUAD datasets containing survival information: TCGA-LUAD, OncoSG-LUAD, GSE68465, and GSE72094 from public data repositories. The TCGA-LUAD dataset was sourced from The Cancer Genome Atlas (TCGA) project and downloaded from the cBioPortal database (https://www.cbioportal.org/ (accessed on 19 March 2025.)). This dataset includes 515 LUAD samples, providing tissue RNA sequencing data, clinical information, and overall survival data. The OncoSG dataset was also obtained from the cBioPortal database [30] and consists of RNA sequencing profiles and clinical data for 169 LUAD patients. The GSE68465 [31] and GSE72094 [32] datasets were downloaded from the Gene Expression Omnibus (GEO). GSE68465 contains gene expression microarray data, clinical information, and pathological data from 443 LUAD patients [31]. GSE72094 includes gene expression data derived from Affymetrix microarrays and clinical information for 442 LUAD patients [32]. Detailed metadata for these four datasets is provided in Supp. Table S2.

### 2.2. scRNA-seq Data Preprocessing

The scRNA-seq data were processed with the Seurat package (version 5.1.0) in R. Initially, we excluded genes that were detected in less than 0.1% of total cells. We quantified the expression of mitochondrial genes and filtered out cells where mitochondrial gene expression exceeded 20%. The cells that had expressed fewer than 200 or more than 5000 genes were also removed. The dataset was normalized using log transformation with a scale factor of 10,000 (scale factor = 1 × 10^4^). Then, we centered the expression values for each gene to have a mean of zero and limited the maximum value to 10 for scaled expression values using the ScaleData function in Seurat by setting the parameters: do.scale = FALSE, do.center = TRUE, scale.max = 10.

### 2.3. Cell Clustering and Cell Lineage Analysis

We selected the top 3000 genes that displayed the highest variability for cell clustering analysis. Dimensionality reduction was performed via principal component analysis (PCA), and the first 20 principal components were selected based on an Elbow plot analysis. The 20 principal components with a resolution of 0.2 were used to identify cell neighbors and generate broad cell clusters. Subsequently, when focusing on specific cell subsets (such as immune, stromal, and epithelial cells), the resolution was lowered to 0.01 to achieve finer-grained clustering. The clustering results were visualized using the uniform model approximation and projection (UMAP) [33].

Purity and entropy were calculated to assess the homogeneity of stage-specific clusters in malignant and non-malignant epithelial cells. Purity quantifies the dominance of a single cell type in a cluster and is computed as follows:

(1) $$\mathrm{Purity}=\frac{1}{N}\sum_{i}\max_{j}\left|c_{i}\cap t_{j}\right|$$

where *N* is the total number of cells, $c_{i}$ is the number of cells in the *i*th cluster, and $t_{j}$ is the count of cells in the *j*th cluster represents true stage information. Purity ranges from 0 (no homogeneity) to 1 (complete homogeneity). Entropy measures the uncertainty within a cluster and is calculated as:

(2) $$S=-\sum_{j=1}^{C}p_{j}\log_{2}\left(p_{j}\right)$$

where C is the number of unique cell types and $p_{j}$ is the proportion of cells of type j. Entropy values range from 0 (complete homogeneity) to 1 (maximum heterogeneity). Higher purity and lower entropy indicate more homogeneous clusters.

We applied the ’FindMarkers’ function in the Seurat package to identify differentially expressed genes (DEGs) between various cell clusters using a Wilcoxon rank-sum test. A minimum percentage (min.pct) threshold of 0.25 was applied. Genes with adjusted *p*-value less than 0.01 and $\log_{2}\left(FC\right)$ greater than 1 were considered differentially expressed.

The trajectory of the cell lineage was constructed using Monocle2 [34]. The raw count matrix, phenotype data, and feature data were extracted from the corresponding Seurat object to generate the ‘newCellDataSet’ object. The data were normalized using ‘estimateSizeFactors()’, and gene expression dispersion was estimated with ‘estimateDispersions()’. A set of highly variable genes, previously identified in Seurat via the ‘VariableFeatures’ function, was used to define the ordering filter with the ‘setOrderingFilter’ function. Dimensionality reduction and trajectory construction were performed using the DDRTree method, and cells were subsequently arranged along a pseudotime axis using ‘orderCells’. The resulting cell trajectories were visualized with ‘plot_cell_trajectory’, highlighting distinct cell states and disease stages.

### 2.4. Copy Number Variation from Single Cell RNAseq Data

To distinguish malignant cells from non-malignant cells in tumor tissues, we utilized a method developed by Nayoung Kim et al. [28] to identify aberrant copy number variations based on perturbations in chromosomal gene expression. First, normal cells were added to tumor cells to ensure that putative malignant cells comprised less than 20% of the total cell population. Genes expressed in fewer than ten cells or with a mean expression of less than 0.1 across all cells on the log2 scale were then filtered out. Gene expression was standardized to Z-scores, scaled from −3 to 3. We sorted the genes by chromosomal location and calculated the CNV signals using a window size of 100 genes. The CNV signal was then adjusted using centered values across all genes. Finally, the CNV signal was summarized using the mean squares of estimates across all windows and the correlation of the CNV for each window with the mean of the top 5% of cells. Tumor cells with a mean square larger than 0.02 or a correlation larger than 0.2 were considered malignant.

InferCNV (version 1.18.1) [35] R package was employed to establish CNV profiles in tumor epithelial cells. Following cluster identification, the scRNA-seq data were prepared for inferCNV by sorting genes according to their genomic locations on chromosomes. As the dataset included only malignant cells, a reference group was not created. The parameters used for the InferCNV run() were cutoff = 1, denoise = FALSE, HMM = FALSE, and cluster_by_groups = TRUE.

### 2.5. LUAD Patient Subgroups

The bulk RNA sequencing and clinical data of LUAD patients in the TCGA (The Cancer Genome Atlas) [23] project were downloaded from cBioPortal [36]. The expression profile was normalized using the $\log_{2}\left(x+1\right)$ transformation. ComplexHeatmap package in R [37] was employed to perform hierarchical cluster analysis using the expression levels of stage-specific DEGs derived from epithelial cells. Euclidean distance with the Ward.D method was adopted for the clustering analysis. The column dendrogram of the hierarchical clusters represented LUAD tissue sample subgroups, while the row dendrogram organized the genes into three subsets. The enriched pathways and biological processes associated with the gene subsets were identified using the enrichKEGG() and enrichGO() functions from the clusterProfiler package in R. The *p*-values were calculated using the hypergeometric distribution and adjusted with the Benjamini-Hochberg (BH) method.

The Kaplan-Meier curves were plotted for each patient subgroup using the R survival package. Patients without stage information were excluded from the survival analysis. The logarithmic ranking test was used to evaluate the significance of the survival rate difference among the patient subgroups.

The composition of stromal and immune cells within LUAD tissue samples was assessed using the ESTIMATE method [38], while tumor purity was calculated using the PUREE method [39]. Both approaches relied on transcriptomic data obtained by RNA sequencing.

### 2.6. Gene Expression Regulatory Networks

We utilized the pySCENIC [40] pipeline to infer gene regulatory networks from processed scRNA-seq data. In the initial step, we employed the ‘grnboost2’ method to identify transcription factor (TF) and target co-expression modules. To reduce the stochasticity of ‘grnboost2’, we repeated this step 100 times, each with a different random seed, generating 100 adjacency files containing TF-target pairs with their importance values. We then retained those TF-target pairs that appeared in at least 80 out of the 100 runs and calculated their importance scores as the average importance across these retained occurrences. We then refined these pairs based on the enrichment of TF binding motifs to infer potential regulons for TFs and their direct targets. The genomic region considered for binding motif searching included 500 base pairs upstream and 100 base pairs downstream of the transcription start site, which defined the promoter region. Subsequently, we quantified the activity of each regulon using the area under the recovery curve [40]. The stage-specific specificity of each regulon was calculated based on Jensen-Shannon divergence [40]. Finally, active and stage-specific regulons containing at least one DEG were assembled into regulatory networks and visualized using Cytoscape.

The interactions in the STRING database were selected based on evidence from experiments, databases, and literature text mining to investigate their connections with the key TFs.

### 2.7. Intercelluar Communication Networks

The cell-cell communication networks were constructed using the CellChat R package [41]. The gene expression data from the scRNA-seq dataset were mapped to a signaling molecular database that consists of interactions among ligands, receptors, and cofactors [41]. Communication networks between cell types were established using mass action models, differential expression analysis, and statistical tests. For a better comparison between early and late stages, we excluded endothelial cells and mast cells because of the absence of data for these two cell types in the late stage. Separate CellChat objects were created for the early and latestage datasets using the normalized data from the Seurat object. We then merged the two CellChat objects using the mergeCellChat() function, allowing for a direct comparison of their intercellular communication networks. To assess differences in the overall number and strength of interactions, we utilized compareInteractions() and netVisual_diffInteraction() to visualize changes in network edges. We also employed netVisual_heatmap() to examine these differences, highlighting variations in incoming and outgoing signaling for each cell type. Additionally, we used rankNet() to evaluate and rank the significance of different signaling pathways. The significance of these signaling pathways was evaluated through a permutation test by randomly permuting cell labels. The random permutation was repeated 100 times, after which the *p*-value was calculated, with p<0.05 considered significant.

## 3. Results

### 3.1. The Cellular Landscape of LUAD

The single-cell RNA sequencing dataset (GSE131907) used in our study was generated from 11 LUAD adjacent normal tissue samples and 15 LUAD tumor tissue samples from eight Stage I, one Stage II, two Stage III, and four Stage IV patients [28]. After removing low-quality and non-expressed genes, a total of 98,504 cells consisting of 55,655 from tumor tissues and 42,849 from normal tissues, and 18,900 genes were retained for further analysis.

**Figure 1:**
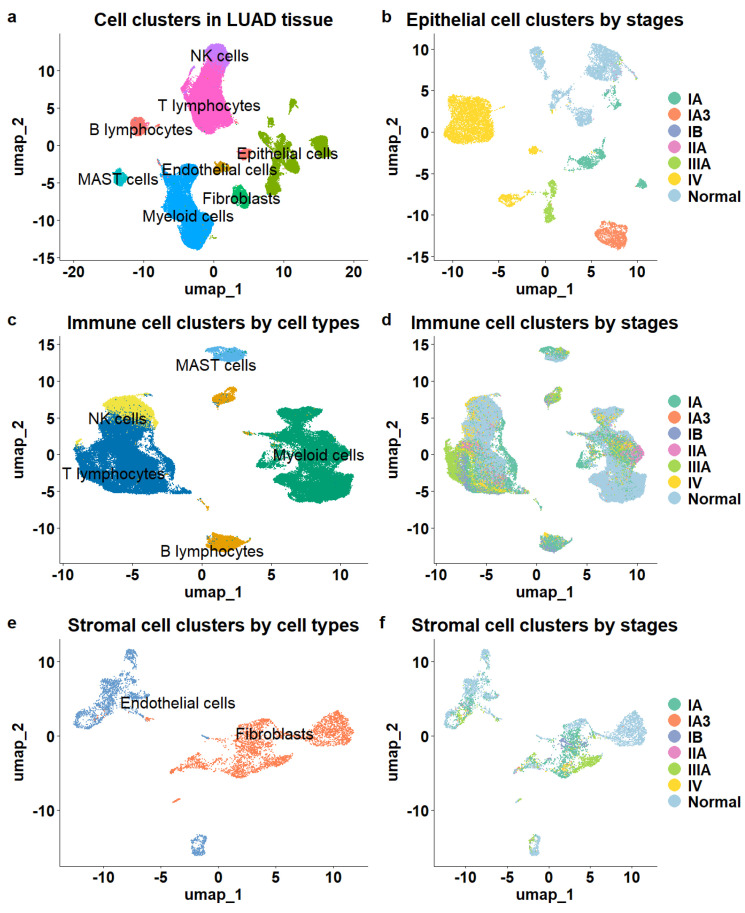
Overview of cell populations in the tumor microenvironment of LUAD. (**a**) The cellular composition of the TME. (**b**) Epithelial cells color-coded by stage demonstrated clear stage separation. (**c**) Immune cells color—coded by cell type showed clear separation. (**d**) Immune cells color—coded by stage lacked stage separation. (**e**) Stromal cells exhibited distinct cell type separation. (**f**) Stromal cells lacked stage—specific distinction.

A total of eight major cell types were identified (Figure 1a), including 16,046 epithelial cells, 5468 stromal cells composed of endothelial cells and fibroblasts, and 76,990 immune cells. The immune cell population comprised 6407 B lymphocytes, 32,591 T lymphocytes, 2888 mast cells, 26,693 myeloid cells, and 8411 natural killer cells (NK). The different cell types were clearly separated, as illustrated in the UMAP (Figure 1a).

Next, we mapped tumor stage information to cell clusters and observed distinct patterns among epithelial, immune, and stromal cells. The epithelial cells demonstrated clear stage-specific clustering, showing significant separation between cells derived from various stages of LUAD tissue samples (Figure 1b) and those from normal tissue samples. In contrast, while stromal and immune cells exhibited distinct separation based on cell subtype (Figure 1c,e), they did not show significant distinctions among cells from different stages or normal tissues. Immune and stromal cells from various stages of LUAD and normal tissues were intermixed within the clusters (Figure 1d,f), indicating a lack of clear stage-specific expression profiles for these cell types.

To determine whether the same phenomenon occurs in other LUAD datasets, we examined an independent patient cohort (GSE127465) [29]. The single-cell RNA sequencing data for this cohort were generated from tumor tissue samples of five LUAD patients and included 2199 epithelial cells, 26,024 immune cells, and 1090 stromal cells. Among these, 9953 cells were from stage I, 9681 from stage III, and 9679 from stage IV. Consistent with the previous dataset, immune and stromal cells showed no distinct separation across different stages, while only epithelial cells formed stage-specific clusters (Supp. Figure S1).

Our findings align with the known roles of epithelial cells in cancer development [42]. In malignant neoplasms, epithelial cells undergo molecular alterations that lead to uncontrolled proliferation and loss of differentiation. Additionally, epithelial cells can acquire mesenchymal traits through processes like epithelial-mesenchymal transition (EMT), which are closely associated with tumor progression and metastasis. Therefore, genes that differentiate epithelial cells of different tumor stages could offer more precise cellular markers corresponding to LUAD progression.

### 3.2. Cellular Gene Markers Associated with LUAD Progression

As epithelial cells from tumor tissues may include residual non-malignant cells, we separated definitive malignant cells from non-malignant ones based on CNV estimation (Section 2). Out of the 12,410 epithelial cells extracted from tumor tissues, we identified 8460 (68.17%) as malignant, including 3572 cells in stages I, II and III and 4888 stage IV cells in the late stage.

**Figure 2:**
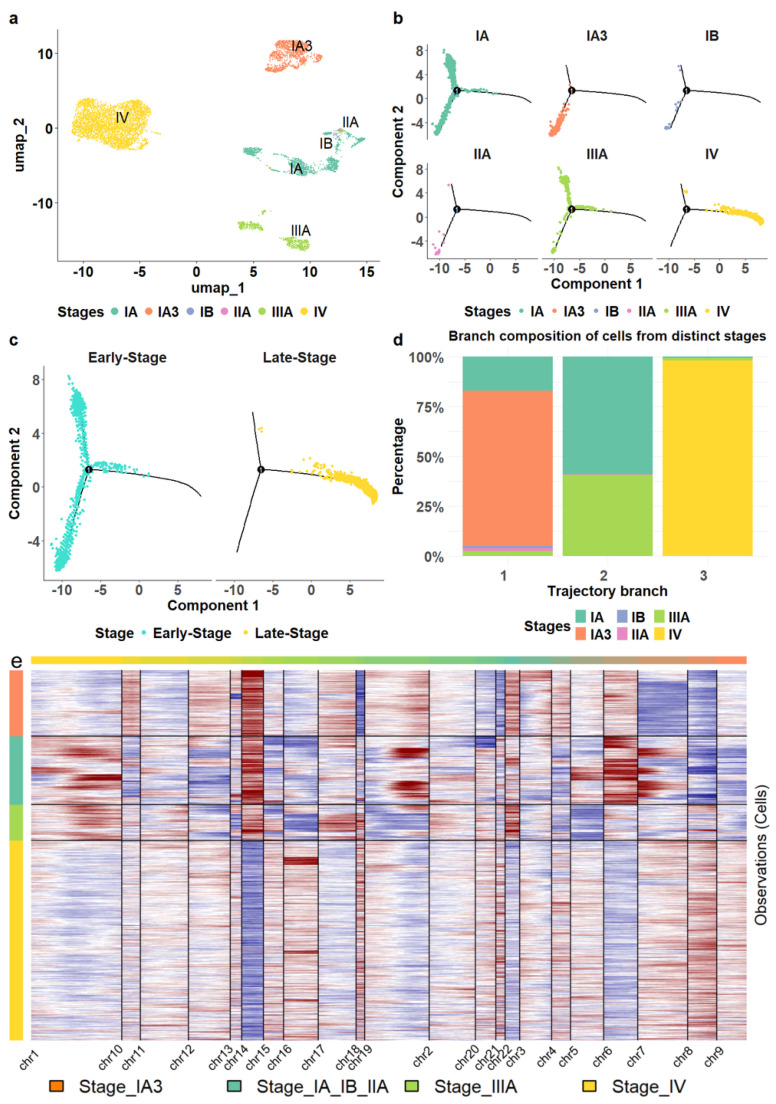
Transcriptomic alterations of epithelial cells during LUAD progression. (**a**) The malignant epithelial cells formed stage—specific clusters. (**b**) The location of epithelial cells from different stages in cell trajectory. (**c**) The comparison of the trajectory locations of epithelial cells from stage IV versus those from earlier stages. (**d**) The composition of epithelial cells in each trajectory branch. (**e**) The epithelial cells from stage IV exhibited distinct copy number variation patterns compared to those from earlier stages.

The malignant epithelial cells formed four cell clusters with varied compositions (Figure 2a). The three clusters were homogeneous, consisting of epithelial cells from stages IV, IIIA, and IA3, respectively (Figure 2a). The heterogeneous cluster comprised a mix of different stages, including 1348 cells from stage IA (89.8%), 19 cells from stage IB (1.3%), 28 cells from stage IIA (1.9%), and 105 cells from stage IIIA (7%). Interestingly, we observed that non-malignant epithelial cells also displayed a stage-specific pattern; however, stage-specific clusters of non-malignant epithelial cells demonstrated lower levels of homogeneity, as indicated by purity and entropy measurements (see Section 2), compared to malignant epithelial cells (Supp. Figure S2). Only malignant cells were retained for further analysis.

Moreover, we reconstructed the cell lineage of epithelial cells. For comparison, we also established cell trajectories for stromal and various immune cells, including T cells, B cells, myeloid cells, and NK cells (Supp. Figure S3). Unlike the distinct separation observed in the trajectory of epithelial cells (Figure 2b), stromal and immune cells from different stages were distributed throughout all branches without clear delineation (Supp. Figure S3), consistent with the patterns observed in the cell clustering results.

We found that the epithelial cell trajectory was closely aligned with tumor progression. Stage IA3 cells were positioned in the bottom left branch of the trajectory, while Stage IA cells were predominantly found on the lower left and upper left branches (Figure 2b). Stage IIIA cells were located mainly on the upper left, some at the beginning of the right branch. The right branch started with a small number of stage III cells, transitioning to predominantly stage IV cells. This configuration illustrates a clear progression pattern through the branches (Figure 2c,d) and a distinct separation of stage IV epithelial cells from those in the earlier stages. Hence, we classified stages I, II, and III as early stages while considering stage IV as a late stage in this study.

We also investigated copy number variations in epithelial cells using InferCNV [43], a software tool that infers genomic amplifications and deletions from scRNA sequencing profiles. Our analysis revealed that epithelial cell transcriptomes in stage-specific clusters displayed unique amplification and deletion patterns on most chromosomes, chromosomes 14 and 8 exhibiting the most significant contrasts between the late and early stages (Figure 2e). In particular, chromosome 14 showed substantial deletions in cells from stage IV, while large amplifications were observed in cells from earlier stages. Conversely, chromosome 8 exhibited remarkable amplification in stage IV cells but showed deletions in the earlier stages.

Together, our results suggested that epithelial cells from stage IV LUAD exhibited significant transcriptional differences compared to those in earlier disease stages (I–III). To further investigate these stage-specific alterations, we conducted a differential expression analysis, comparing the epithelial cell populations isolated from earlier stage (I–III) tumors to those obtained from stage IV samples. This analysis identified the top 55 genes that were significantly upregulated (adjusted *p*-value < 0.01, $log_{2}$(fold change) > 1) in the stage IV epithelial cells. In contrast, these genes did not show significant expression changes in epithelial cells derived from normal tissues or non-malignant epithelium when comparing stage IV to earlier stages (Supp. Figure S4). Given the central role of epithelial cells in LUAD progression, we hypothesized that these stage-specific differentially expressed genes (DEGs) could serve as robust cellular markers characterizing disease progression. To evaluate the validity and clinical relevance of these putative markers, we examined their expression patterns across multiple independent LUAD patient cohorts.

### 3.3. Clinical Implications of Cellular Marker Genes

**Figure 3:**
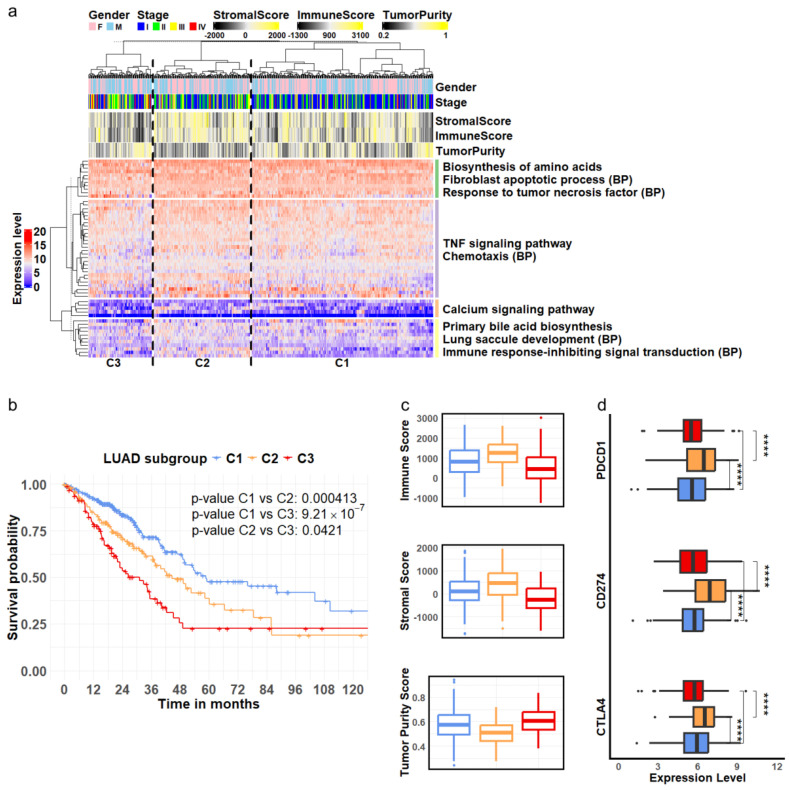
LUAD subgroups defined by cellular markers associated with disease progression. (**a**) Hierarchical clustering analysis of RNA—seq expression of 55 genes derived from epithelial cells revealed three LUAD subgroups, C1, C2, and C3. These genes were separated into four clusters enriched in distinct pathways and biological processes (BPs) related to tumor development. (**b**) Among the three identified subgroups, C1 (blue), C2 (orange) and C3 (red), Kaplan—Meier survival curves indicate that patients in subgroup C3 (red) had the poorest survival outcomes, while those in subgroup C1 (blue) exhibited the best survival. (**c**) The C3 (red) subgroup showed the highest tumor purity, accompanied by the lowest levels of stromal and immune cell content. (**d**) The three genes encoding immune inhibitors were expressed at significantly higher levels in subgroup C2 (orange) compared to subgroups C1 (blue) and C3 (red). (**** p<0.0001).

We first analyzed a cohort of LUAD patients from the TCGA project. Hierarchical clustering of the expression levels of the identified stage-specific DEGs derived from epithelial cells classified the LUAD patients into three distinct subgroups: C1, C2, and C3, as illustrated in the column dendrogram of Figure 3a. Concurrently, the genes were organized into four groups according to the row dendrogram (Figure 3a), with each group exhibiting significant enrichment in biological processes and pathways relevant to tumor biology [44,45,46,47,48,49]. The statistical significance of these functional enrichments was assessed using *p*-values calculated by the hypergeometric distribution and adjusted using the Benjamini-Hochberg (BH) method (Supp. Table S3).

Survival analysis revealed significantly distinct outcomes among the three patient subgroups. Patients classified into subgroup C1 demonstrated the best survival rates, while those in subgroup C3 experienced the poorest outcomes (Figure 3b). To further validate the clinical relevance of these stage-specific gene signatures, we evaluated their performance in three independent LUAD cohorts: OncoSG, GSE68465, and GSE72094. The results showed the consistency of these gene markers in segregating patients into subgroups with varying survival outcomes (Supp. Figure S5).

Furthermore, we investigated the TME composition across the three patient subgroups identified through our clustering analysis. We assessed the immune and stromal cell content using the ESTIMATE algorithm [38] and evaluated tumor purity using the PUREE method [39]. The results indicated that tumor samples in subgroup C3 had the lowest stromal cell composition, the lowest level of immune infiltration, and the highest tumor purity compared to subgroups C1 and C2 (Figure 3c, ANOVA test, p<0.05). Interestingly, while subgroup C2 exhibited the highest immune infiltration, its patients had lower survival rates than those in subgroup C1. To further explore the potential mechanisms underlying these survival differences, we examined the expression levels of known immune checkpoint genes. We observed significantly higher expression of *PDCD1* (encoding PD-1), *CD274* (encoding PD-L1), and *CTLA4* in subgroup C2 (Figure 3d). PD-1 is an inhibitor of T-cell activation [50], while PD-L1 can downregulate immune responses by interacting with PD-1 [51]. Similarly, CTLA-4 acts as a negative regulator of T-cell activation [52]. The elevated expression of these immune checkpoint molecules in subgroup C2 suggested potential tumor evasion of immune surveillance and T-cell exhaustion, which may result in a negative impact on patient survival in this subgroup.

Collectively, our results indicated that the DEGs derived from epithelial cells captured critical molecular characteristics of LUAD progression and suggested that epithelial cells may significantly influence the biological behavior of other cell types within the TME.

### 3.4. Key Transcription Regulators of Progression-Related Gene Markers

**Figure 4:**
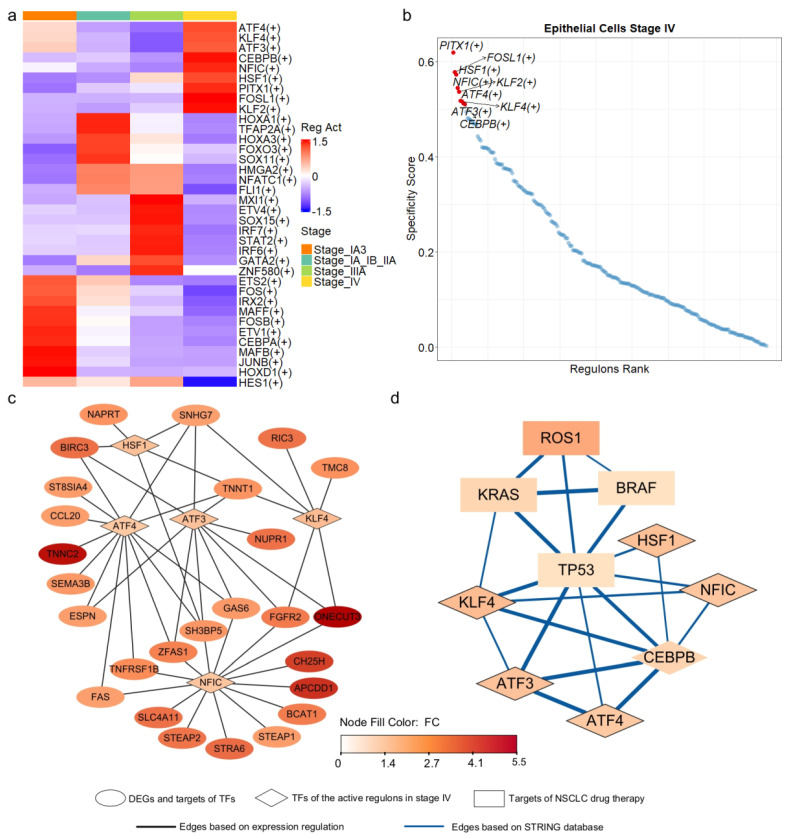
Transcriptional regulatory networks of stage—specific DEGs derived from epithelial cells. (**a**) Active regulons identified in epithelial cells across the four stages of LUAD, with each regulon being active in at least one stage. (**b**) Regulons ranked based on their specificity to stage IV epithelial cells. (**c**) Transcriptional regulatory networks of the DEGs, with TF represented by diamonds and target DEGs represented by ovals. Node color corresponded to fold change, indicating the level of expression variation between late—stage and early—stage epithelial cells. (**d**) Interaction network derived from the STRING database, with known targets of NSCLC drug therapy represented by rectangles and TF represented by diamonds.

To identify the upstream regulators of the 55 progression-related DEGs, we inferred transcriptional regulatory networks in epithelial cells using SCENIC [40]. This method integrates scRNA-seq data with transcription factor (TF) binding information to construct regulatory networks (see Section 2). Among the 1892 human TFs examined, 218 TFs and their downstream targets formed co-expression modules, with target gene promoters enriched for binding motifs corresponding to their respective TFs within each regulon. We assessed regulon activity using the area under the recovery curve and calculated stage specificity based on Jensen-Shannon divergence [40]. This analysis identified 36 regulons active in one or more cell types (Figure 4a), with nine regulons specific to stage IV (Figure 4b). We then assembled networks from active and stage-specific regulons containing at least one DEG.

In the identified regulatory networks, 32 of the 55 DEGs were classified as target genes, while *PITX1*, one of the DEGs, served as a transcription factor within the network. *PITX1* can activate the tumor suppressor gene *TP53* by binding to its promoter region [53]. Among the 32 target DEGs, 26 were regulated by five transcription factors (TFs): *ATF3*, *ATF4*, *HSF1*, *KLF4*, and *NFIC* (Figure 4c). Notably, these five TFs functioned as regulators within the regulons that were highly active and stage-specific in epithelial cells at stage IV (Figure 4a,b).

*ATF3* and *ATF4*, belonging to the activating transcription factor (ATF) family, are involved in critical cancer biology pathways [54] and mediate cellular responses to stress [55,56]. *HSF1*, a vital regulator of the heat shock response, has been implicated in tumor malignancy and poor prognosis [57]. *KLF4* plays a crucial role in regulating cell growth and differentiation [58], and its low expression has been associated with poor survival outcomes in LUAD [59]. Finally, *NFIC*, a member of the nuclear factor I family, has been implicated in various types of cancer [60,61,62,63].

The five transcription factors (TFs) regulated the expression of 26 out of 55 differentially expressed genes (DEGs) (Figure 4c). Notably, *ATF4* and *NFIC* regulated 13 and 14 of these genes, respectively. To further investigate the functional relevance of the five key transcription factors (TFs), we performed Gene Ontology (GO) and KEGG pathway enrichment analyses on their predicted target genes. The results revealed that these TFs regulate diverse and biologically meaningful processes related to LUAD progression. ATF3 targets were significantly enriched in the fibroblast apoptotic process (*p*.adjust < 0.013) and EGFR tyrosine kinase inhibitor resistance (*p*.adjust < 0.024). ATF4 targets were associated with TNF signaling (*p*.adjust < 0.00011), cytokine–receptor interactions (*p*.adjust < 0.034), and platinum drug resistance (*p*.adjust < 0.034). NFIC targets were enriched in tumor necrosis factor-mediated pathways (*p*.adjust < 0.038) and lung epithelium development (*p*.adjust < 0.040). KLF4 targets were involved in pathways related to epithelial proliferation and lung morphogenesis (*p*.adjust < 0.060). HSF1 targets showed significant enrichment in processes such as programmed necrotic cell death (*p*.adjust < 0.050) and nucleotide salvage (*p*.adjust < 0.041). Furthermore, incorporating interactions from the STRING database, we found that four genes, *TP53*, *KRAS*, *ROS1* and *BRAF*, the known targets of NSCLC drug therapy, were directly or indirectly interconnected with these TFs (Figure 4d). Collectively, our results, corroborated by multi-layered evidence from literature and databases, suggest that these TFs may function as critical molecular drivers in LUAD progression.

### 3.5. Cellular Communication Alterations in Response to Tumor Progression

**Figure 5:**
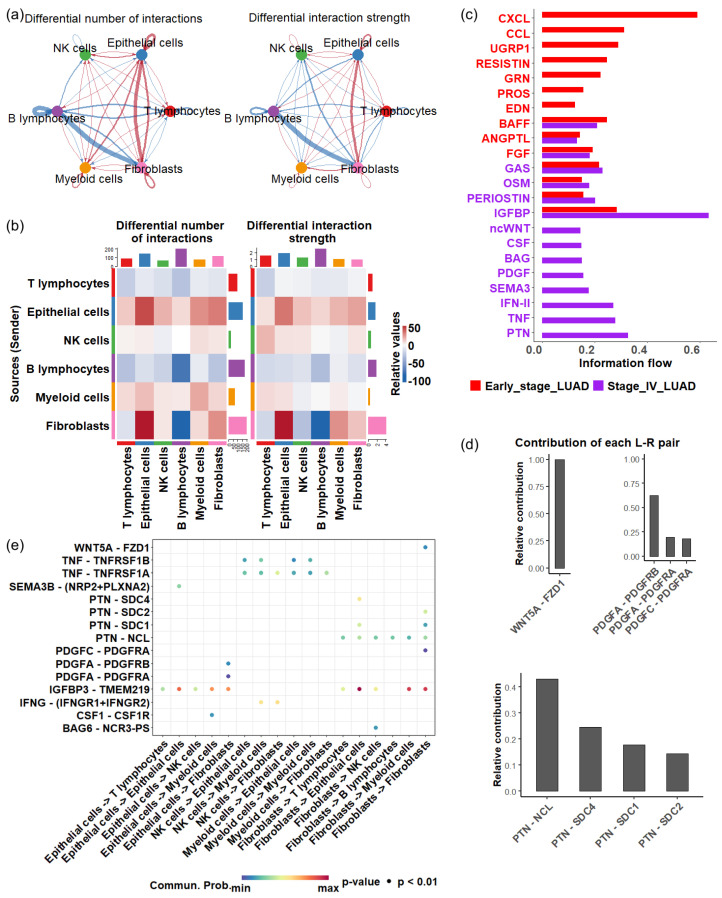
Cell—cell communications in early and late stage of LUAD. (**a**) Differential cell—cell communication number (left) and strength (right). Blue arrows represent a decrease in the number or strength of interactions in the late stage of LUAD compared with the early stage of LUAD, while red arrows represent an increase in interactions in the late stage of LUAD. (**b**) Differential cell—cell communication number (left) and strength (right). (**c**) Significant signaling pathways in the early and late stages of LUAD (*p* < 0.05). The active pathways in the early stage are represented by red bars, while those in the late stages are shown in purple bars. (**d**) Contribution of ligand—receptor pairs in the ncWNT pathway (upper) and PTN pathway (bottom). (**e**) Significant ligand—receptor interactions (*p* < 0.01), represented by dots, contribute to cellular communication in late—stage LUAD.

To understand how cellular interactions evolve during disease progression, we established cell-cell communication networks among various cell types in the TME. When comparing stage IV LUAD with earlier stages, we found a decrease in both the total number and strength of cellular signaling originating from T cells to other cell types (Figure 5a,b). A similar decline was observed for B cells (Figure 5a,b), indicating an overall reduction in immune response during the late stage of LUAD.

In contrast, there was a substantial increase in cellular communication originating from epithelial cells in stage IV, characterized by a greater number of interactions and enhanced interaction strength (Figure 5a,b).In particular, a significant rise in communications between epithelial cells and fibroblasts was observed (Figure 5a,b). Cancer-associated fibroblasts are known to promote angiogenesis, metastasis, immune evasion, and drug resistance [64,65,66]. Additionally, we detected heightened intercellular crosstalk between myeloid cells and epithelial cells, and the communication originating from myeloid cells to T cells also increased in stage IV. Research indicates that myeloid-related innate immunity can facilitate tumor progression by affecting T-cell activity [67]. Together, these results suggest that the complex cellular interplay centered around epithelial cells may significantly impact how the TME mediates LUAD development.

Next, we investigate the activity of signaling pathways that coordinate cell communications during LUAD progression, focusing on secreted signaling. Of 158 secreted signaling pathways, 22 exhibited significant changes (p<0.05) between stage IV and earlier stages. Among the significant pathways, eight were exclusively active in late-stage LUAD, which included ncWNT (noncanonical Wnt pathway), CSF (colony-stimulating factors), BAG (BCL-2-associated athanogene), PDGF (platelet-derived growth factor), SEMA3 (semaphorin 3), IFN-γ (interferon gamma), TNF (tumor necrosis factor) and PTN (pleiotrophin) (Figure 5c).

The activation of the ncWNT pathway enhances cell migration and contributes to metastatic behavior. Dysregulation of this pathway promotes the EMT, allowing tumor cells to invade surrounding tissues and spread through the bloodstream [68]. The EMT mechanism can also cause drug resistance [69]. In the ncWNT pathway, the WNT5A → FZD1 interaction represented the dominant ligand-receptor pairing (Figure 5d(top left panel)).

PDGF signaling pathway involves angiogenesis and EMT, promoting tumor metastasis [70,71]. The significant ligand-receptor pairs involved in the PDGF signaling pathway were PDGFA → PDGFRB and PDGFA → PDGFRA, which facilitate signaling from epithelial cells to fibroblasts, whereas the PDGFC → PDGFRA interaction was involved in intracellular signaling in fibroblasts (Figure 5d(top right panel),e).

PTN is a multifunctional growth factor known for its angiogenic properties and role in tumor growth and metastasis [72]. Four key ligand → receptor pairs, including PTN → NCL, PTN → SDC4, PTN → SDC1, and PTN → SDC2, contributed to the activity of the PTN pathway (Figure 5d(bottom panel)), three contributed to the signal of fibroblasts to epithelial cells (Figure 5e) while PTN → SDC2 was involved in intracellular communication in fibroblasts. The PTN → NCL interaction is a primary driver of PTN pathway activity in late-stage LUAD. Nucleolin (NCL) is a protein that is often overexpressed in various cancers [73,74,75,76,77], and inhibiting nucleolin can reduce cancer cell viability [78].

Most ligand-receptor interactions contributing to the unique pathways in late-stage LUAD were observed in the signaling between epithelial cells and fibroblasts (Figure 5e). The enhanced crosstalk between these two populations may play a vital role in shaping the TME, highlighting the central roles of epithelial cells in LUAD progression.

## 4. Discussion

In this study, we developed an integrated computational framework to investigate the cellular mechanisms underlying the development of LUAD. We began by analyzing scRNA-seq transcriptomes of various cell types, including stromal, immune, and epithelial cells, in the TME of early- and late-stage LUAD. Our analysis revealed distinct patterns in cell clustering and trajectory, particularly among epithelial cells, in contrast to stromal and immune cell populations. Late-stage LUAD epithelial cells formed a well-defined cell cluster and lineage branch, clearly distinguishing them from their early-stage counterparts. This separation was not observed in the stromal and immune cells. The results were further validated by the scRNA-seq dataset of an independent cohort of LUAD patients. Thus, we hypothesize that gene signatures differentiating early-stage from late-stage epithelial cells provide precise cellular markers related to LUAD progression and metastatic processes. Our analysis of the TCGA LUAD patient cohort revealed that the expression profiles of these marker genes delineated three distinct LUAD subgroups. Each subgroup showed significantly different survival outcomes and TME compositions.

Among LUAD subgroups, tumor tissue from patients with the poorest survival outcomes exhibited the highest tumor purity, the lowest levels of immune infiltration, and low stromal scores. Previous studies suggested that lower stromal scores are associated with better survival in gastric cancer [79] and colon cancer [80]. A low stromal component may limit blood vessel formation, hindering tumor growth; however, it might simultaneously reduce immune cell infiltration, possibly due to the complex crosstalk between stromal and immune cells within the TME. Moreover, the *TP53* mutation rate was significantly high in subgroup C3. Mutated *TP53* are often linked to aggressive tumor characteristics and poor clinical outcomes [81,82]. Additionally, p53 mutations can lead to chronic inflammation in cancer cells and foster an immunosuppressive environment [83,84]. Interestingly, while subgroup C2 was characterized by the highest levels of immune infiltration, this did not correlate with optimal survival outcomes. We observed elevated expression of genes encoding the immune checkpoint proteins in this subgroup. This suggested the phenomenon of T-cell exhaustion, where persistent exposure to tumor antigens diminishes T-cell functionality, ultimately compromising the immune response [85]. This result aligned with existing literature indicating that despite heightened immune infiltration in some cancer subtypes, the presence of immunosuppressive signals can hinder effective immune responses [86].

We observed that female patients were more prominently represented in the subgroups with favorable survival outcomes and less represented in the subgroup associated with poor survival outcomes (Chi-square test, p<0.052, Supp. Table S4). This finding aligns with the existing literature indicating that female patients with LUAD tend to experience better survival outcomes than their male counterparts [87,88]. In addition, we noted variability in tumor stage distribution among the subgroups; each subgroup contained a mix of samples at various stages. However, in comparisons of stage composition across subgroups, stage I had the highest proportion in subgroup (C1) with the best survival rate, while stage IV had the highest proportion in subgroup (C3) with the poorest survival rate (Supp. Table S5). Thus, these progress-related genes derived from epithelial cells can effectively stratify patients into subgroups with distinct clinical characteristics. Although TCGA RNA-seq data reflected average gene expression across heterogeneous cell populations, our results implied that these epithelial cell-derived genes captured critical aspects of LUAD progression. Furthermore, they demonstrated that epithelial cells could significantly influence the biological behavior of other cell types within the TME.

Our investigation into the cell-cell communication networks within the TME revealed significant changes between early and late-stage lung adenocarcinoma (LUAD). Notably, communications originating from T cells and B cells significantly decreased in late-stage LUAD, suggesting a compromised immune response. This decline in immune cell communication aligns with findings that late-stage tumors often exhibit characteristics associated with immune evasion [89]. Conversely, communication signals from epithelial cells to other cell types in the TME increased in the late stage, including interactions with T cells and B cells. This enhanced signaling includes interactions with T cells and B cells, indicating that epithelial cells may actively modulate the immune landscape within the TME. For instance, secreted factors from epithelial cells can influence T cell differentiation and activation, shaping the immune environment in ways that may either aid or hinder anti-tumor responses [90].

Specifically, we observed significant increases in intercellular communication between epithelial cells and fibroblasts in late-stage LUAD. Fibroblasts are key components of the TME, contributing to its architecture and influencing tumor biology through the secretion of growth factors and cytokines [91,92]. The interplay between epithelial cells and fibroblasts often results in the secretion of pro-inflammatory and pro-angiogenic factors, which can stimulate angiogenesis and further tumor growth [93,94]. The enhanced collaboration between these cell types in late-stage disease may suggest a shift toward a more aggressive tumor phenotype, enabling efficient nutrient supply and persistent tumor cell survival.

Moreover, our study identified multiple signaling pathways activated in late-stage LUAD, such as the ncWNT, PDGF and PTN pathways. The WNT signaling pathway, particularly through its non-canonical (ncWNT) branch, plays a crucial role in epithelial-mesenchymal transition (EMT) [69,95], a dynamic process wherein epithelial cells lose their cell polarity and adhesion properties, gaining invasive and migratory capabilities [96]. The ligand-receptor interactions that significantly contribute to the activity of these pathways represent promising therapeutic targets. For instance, antagonists targeting these late-stage specific pathways may help disrupt the communication that enables tumor survival and immune evasion [97,98]. PTN has been shown to promote tumorigenesis by enhancing cell proliferation and contributing to the tumor microenvironment [99,100], while PDGF plays a critical role in angiogenesis and fibrosis associated with tumor progression [101]. Preclinical models have demonstrated that inhibition of these pathways can reduce tumor growth and improve treatment responsiveness [99,102,103,104]. Additionally, research suggests that combining PTN and PDGF inhibitors with existing therapies, such as chemotherapy and immunotherapy, may produce synergistic effects, enhancing therapeutic efficacy and overcoming resistance mechanisms [105,106,107]. For example, studies have shown that concurrent targeting of PDGF signaling can enhance the effectiveness of anti-PD-1 therapies by modulating the tumor immune microenvironment [108,109].

The 55 stage-specific genes identified in our study demonstrate considerable potential as biomarkers for patient stratification and prognostic assessment in LUAD. Their consistent performance across multiple independent patient cohorts suggests their clinical utility. In addition, five key transcription factors (ATF3, ATF4, HSF1, KLF4, and NFIC) and critical signaling pathways (ncWNT, PDGF, and PTN) represent potential therapeutic targets, paving the way for the development of novel treatment strategies in advanced LUAD. Integrating these molecular insights into clinical workflows could enhance diagnostic precision and guide therapeutic decision making in LUAD treatment. Collectively, our findings, supported by existing literature, underscore the translational potential of targeting these genes and pathways to not only inhibit tumor progression but also to improve clinical outcomes when combined with current treatment regimens.

Recent literature has proposed a broader conceptual framework that characterizes cancer not solely as a genetic disease, but as an ecological pathology [110]. In this perspective, cancer cells are conceptualized as invasive species that interact dynamically with their microenvironment through ecological principles such as mutualism, parasitism, and competition. Our findings of enhanced epithelial-fibroblast crosstalk and altered ligand-receptor signaling in late-stage LUAD align with this ecological model, suggesting that tumor progression results not only from cell-intrinsic genetic alterations but also from evolving ecological interactions within the tumor microenvironment. These interactions may play crucial roles in fostering immune suppression, therapeutic resistance, and metastasis through non-genetic mechanisms.

While our study provides valuable insights into the roles of epithelial cell subpopulations and cell–cell communication dynamics in LUAD progression, future research, including in vitro and in vivo experiments, is essential to validate the functional roles of the identified key transcription factors and signaling pathways.

In conclusion, our work underscores the pivotal role of epithelial cells in driving LUAD progression. The key transcription regulators and signaling pathways identified in this study offer valuable therapeutic targets for developing more effective and precise treatment strategies for LUAD patients.

## Data Availability

GSE131907, GSE127465 and GSE68465 are available in the Gene Expression Omnibus (GEO) database. TCGA breast cancer data are available at the Genomic Data Commons (GDC) Data Porter (https://portal.gdc.cancer.gov/ (accessed on 19 March 2025)). The OncoSG dataset was also obtained from the cBioPortal database.

## Supplementary Materials

The following supporting information can be downloaded at: https://www.mdpi.com/article/10.3390/biomedicines13071606/s1, Figure S1: Cell clustering analysis of LUAD scRNA-seq dataset GSE127465.; Figure S2: Stage-specific cell clustering in malignant and nonmalignant epithelial cells.; Figure S3: Cell trajectories of stromal cells and various immune cell types at different LUAD stages.; Figure S4: Expression of 55-gene signatures across LUAD stages in normal, non-malignant, and malignant epithelial cells; Figure S5: LUAD patient stratification based on the DEGs derived from epithelial cells and survival analysis in independent cohorts.; Table S1: Single-cell RNA sequencing datasets; Table S2: LUAD datasets with survival information; Table S3: Significantly enriched pathways and biological processes; Table S4: Sex information in patient subgroups; Table S5: Stage information in patient subgroups.
