# Supplementary material for: Single-Cell Transcriptomic Analysis Unveils Key Regulators and Signaling Pathways in Lung Adenocarcinoma Progression
Source: Biomedicines. 2025 Jun 30;13(7):1606. doi: 10.3390/biomedicines13071606 (PMC12292084; doi:10.3390/biomedicines13071606)
Supplement: Supplementary file 1 [file biomedicines-13-01606-s001.zip › biomedicines-3654684-supplementary.pdf]

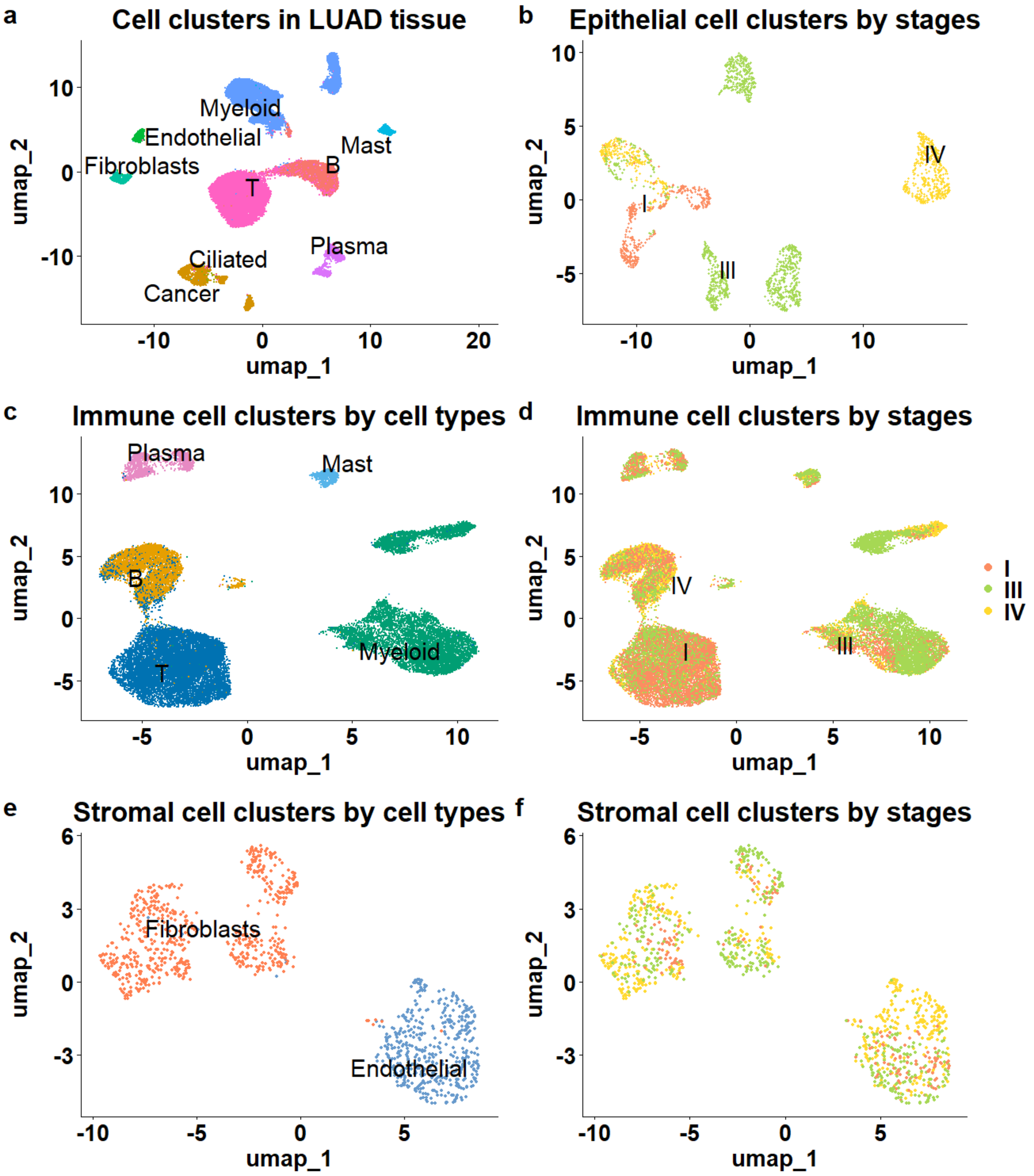

Supp. Figure S1. Cell clustering analysis of LUAD scRNA-seq dataset GSE127465.

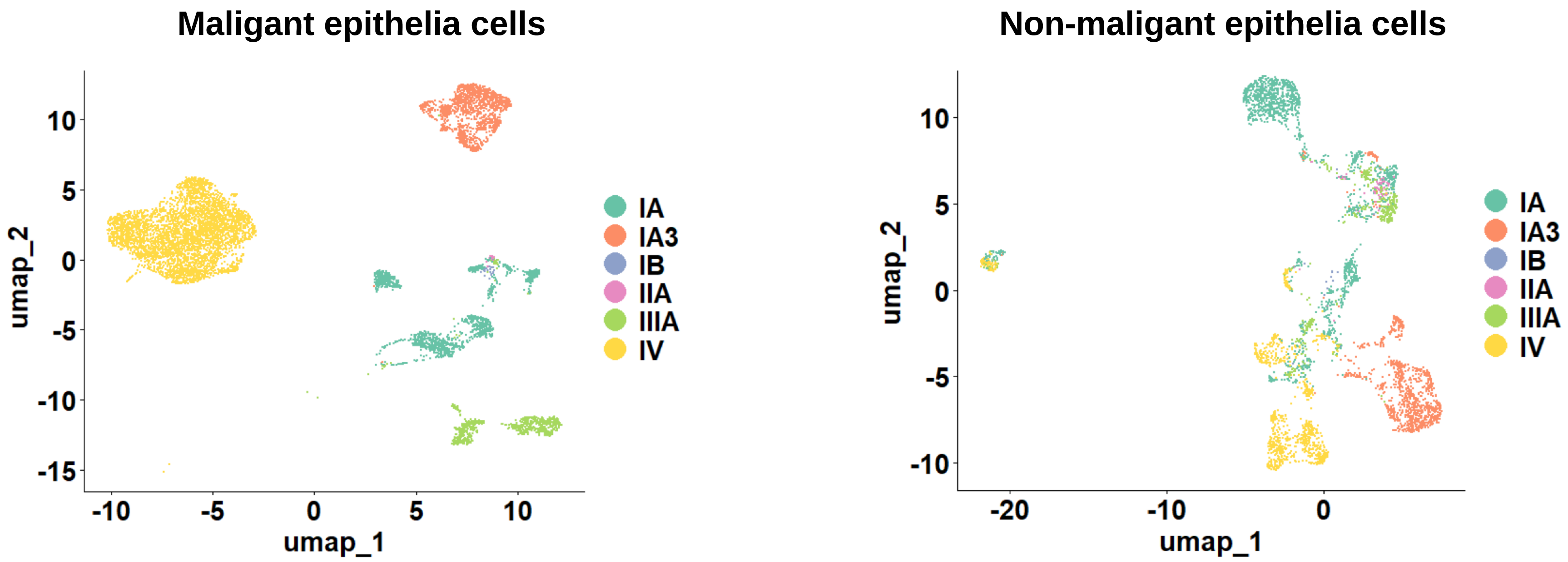

	Purity	Entropy
**Malignant epithelial cells**	0.98	0.045
**Non-malignant epithelial cells**	0.773	0.386

Supp. Figure S2. Stage specific cell clustering in maligant and non-maligant epithelial cells

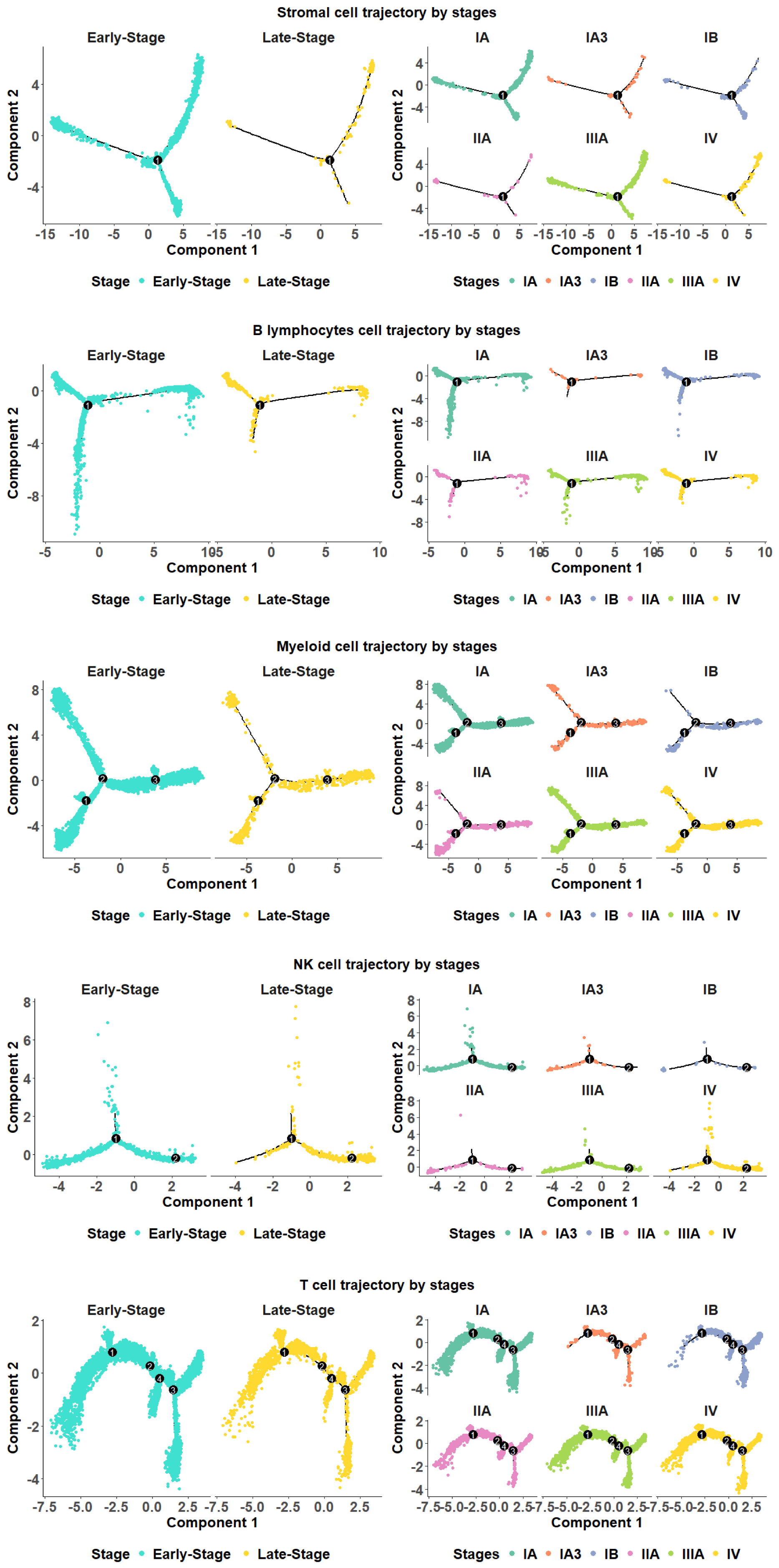

Supp. Figure S3. Cell trajectories of stromal cells and various immune cell types at different LUAD stages.

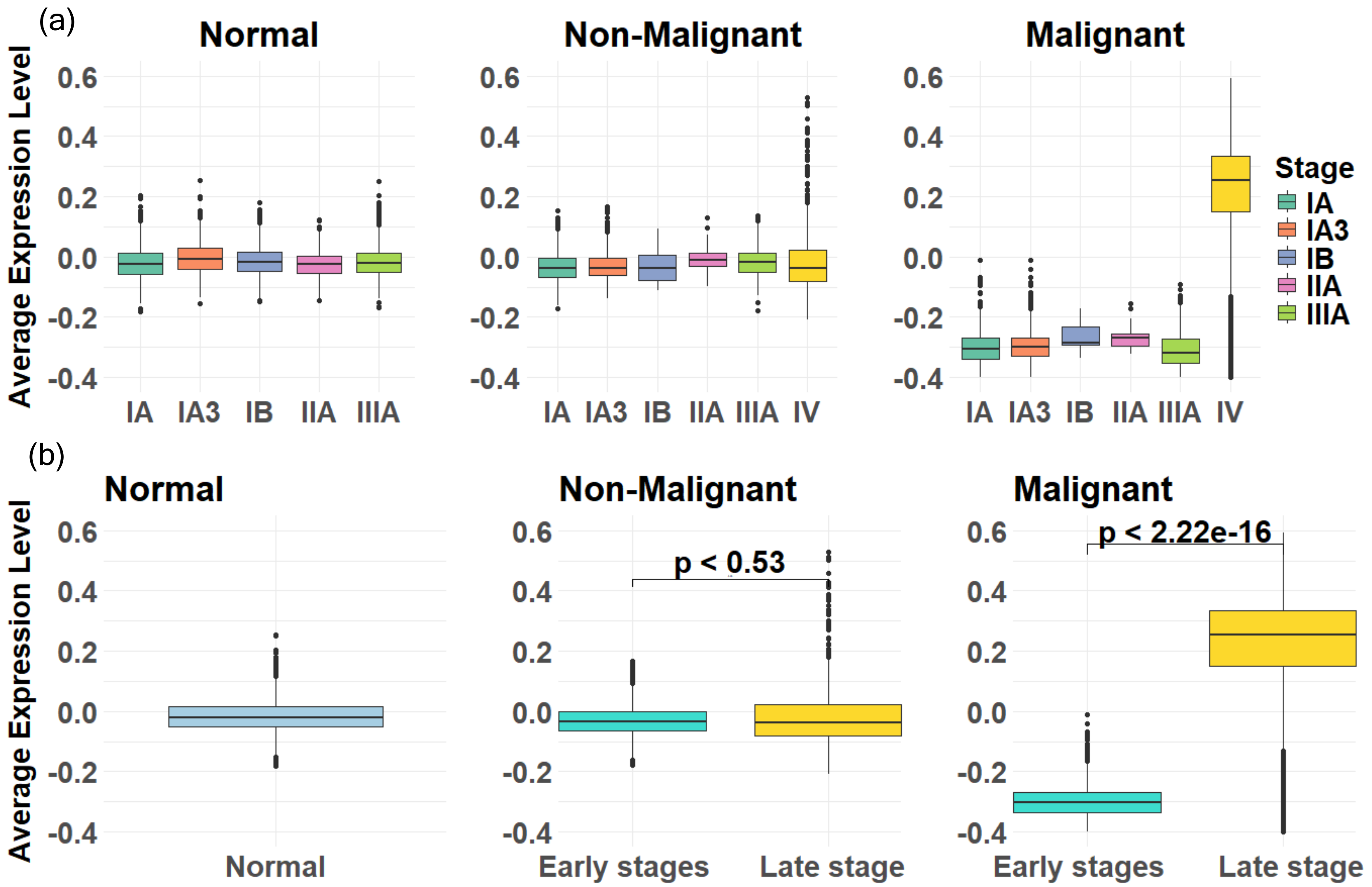

Supp. Figure S4. Expression of 55-gene signatures across LUAD stages in normal, non-malignant, and malignant epithelial cells. (a) Gene expression remains unchanged across stages in normal and non-malignant epithelial cells but shows a marked increase in stage IV malignant epithelial cells. (b) Expression levels are significantly higher in late-stage (IV) malignant cells compared to early stages (I–III) ($p < 2.22 \times 10^{-16}$), with no significant changes in normal or non-malignant cells ($p = 0.53$).

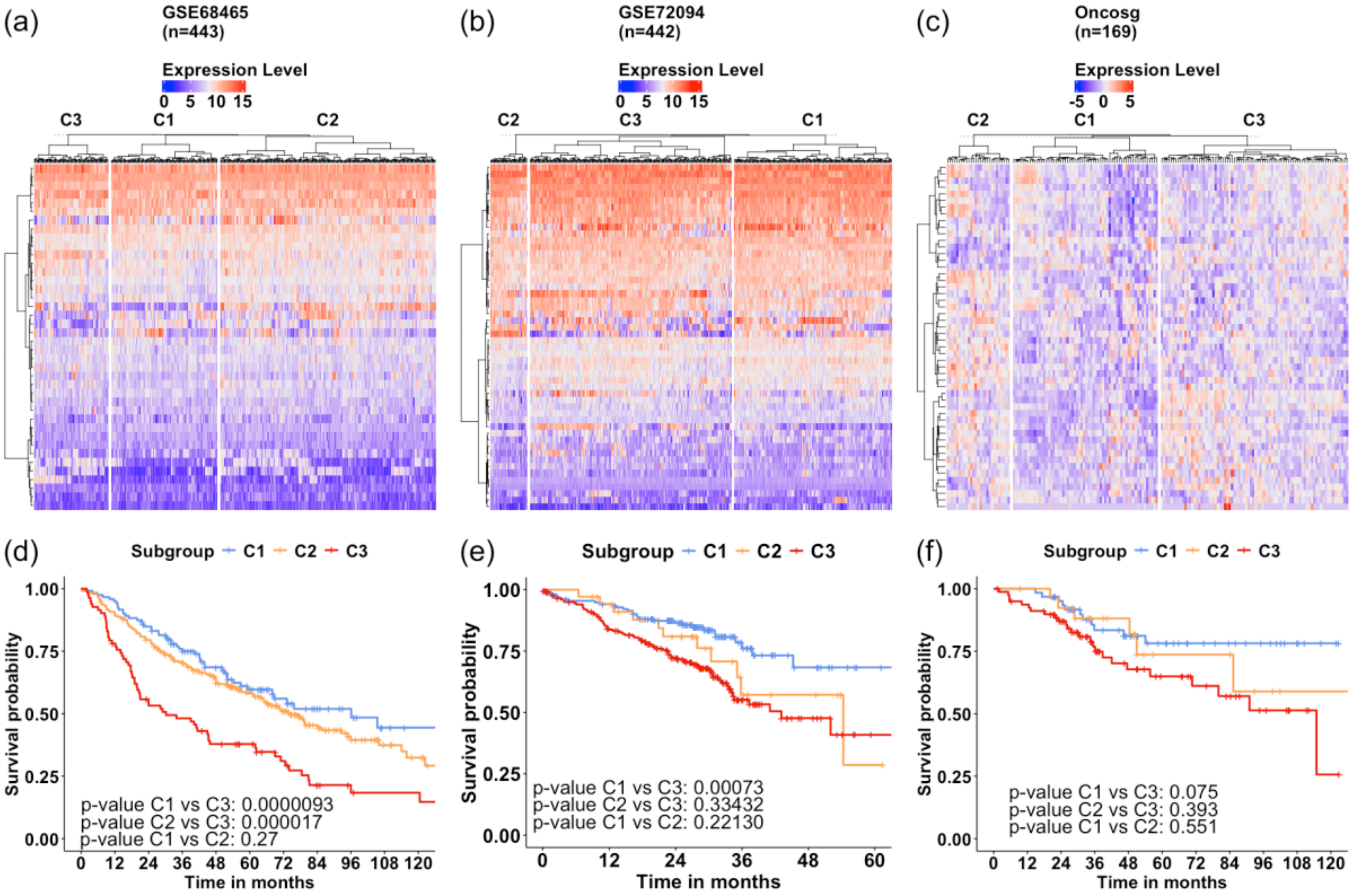

Supp. Figure S5. LUAD patient stratification based on the DEGs derived from epithelial cells and survival analysis in independent cohorts. Patient stratification using progression-related gene markers and survival analysis of the resulting LUAD subgroups. Panel (a) and (d) represent results for the GSE68465 dataset. Panel (b) and (e) represent results for the GSE72094 dataset. Panel (c) and (f) show the results of Oncosg dataset.

Supplementary Table S1: Single-cell RNA sequencing datasets

	GSE131907		GSE127465	
**Age**	**Mean** $\pm\sigma$		**Mean** $\pm\sigma$	
	62.8 $\pm$ 8.05		71 $\pm$ 8.31	
**Sex**	**#Patient**	**#Single-Cell**	**#Patient**	**#Single-Cell**
Male	13 (86.67%)	49192 (88.39%)	2 (40%)	9963 (33.95%)
**Tumor Stage**				
I	8 (53.33%)	30610 (55%)	2(40%)	9953 (33.95%)
II	1 (6.67%)	3813 (6.85%)	0	0
III	2 (13.33%)	9997 (17.96%)	2(40%)	9681 (33.03%)
IV	4 (26.67%)	11235 (20.19%)	1(20%)	9679 (33.02%)

Supplementary Table S2: LUAD datasets with survival information

**Category**	**TCGA-LUAD**	**OncoSg-LUAD**	**GSE68465**	**GSE72094**
**Age**				
Mean $\pm$ σ	65.37 $\pm$ 9.94	64 $\pm$ 9.47	64.42 $\pm$ 10.10	69.30 $\pm$ 9.33
**Sex**				
Male	238 (46.21%)	75 (44.38%)	223 (50.34%)	202 (45.07%)
**Tumor stage**				
I	275 (54.23%)	102 (61.08%)	279 (63.10%)	265 (64.05%)
II	122 (24.08%)	30 (17.96%)	95 (21.50%)	69 (16.67%)
III	84 (16.56%)	31 (18.56%)	68 (15.39%)	63 (15.21%)
IV	26 (5.13%)	4 (2.39%)	0	17 (4.11%)

Supplementary Table S3: Significantly enriched pathways and biological pro- cesses

Description	p value	p-adjust
Biosynthesis of amino acids	0.001 55	0.0373
Fibroblast apoptotic process	0.000 082	0.0249
Response to tumor necrosis factor (BP)	0.000 286	0.0349
TNF signaling pathway	0.000 074	0.005 20
Chemotaxis (BP)	0.000 324	0.0612
Calcium signaling pathway	0.0586	0.0684
Primary bile acid biosynthesis	0.005 96	0.0417
Lung saccule development (BP)	0.005 29	0.0946
Immune response-inhibiting signal transduction (BP)	0.005 82	0.0946

Supplementary Table S4: Sex information in patient subgroups

Sex	C1	C2	C3
Female	156	80	41
Male	118	65	55

Supplementary Table S5: Stage information in patient subgroups

**Stage**	**C1**	**C2**	**C3**
Stage I	169 (**62.8%**)	66 (46.2%)	40 (42.1%)
Stage II	59 (21.9%)	43 (30.1%)	20 (21.1%)
Stage III	30 (11.1%)	28 (19.6%)	26 (27.4%)
Stage IV	11 (4.1%)	6 (4.2%)	9 (**9.5%**)
Total	269	143	95
